# Trends in mental health inequalities for people with disability, Australia 2003 to 2020

**DOI:** 10.1177/00048674231193881

**Published:** 2023-08-22

**Authors:** Glenda M Bishop, Anne Marie Kavanagh, George Disney, Zoe Aitken

**Affiliations:** Disability and Health Unit, Centre for Health Equity, Melbourne School of Population and Global Health, The University of Melbourne, Melbourne, VIC, Australia

**Keywords:** Disability, mental health, health inequalities, time series

## Abstract

**Objective:**

Cross-sectional studies have demonstrated that people with disability have substantial inequalities in mental health compared to people without disability. However, it is not known if these inequalities have changed over time. This study compared the mental health of people with and without disability annually from 2003 to 2020 to investigate time trends in disability-related mental health inequalities.

**Methods:**

We use annual data (2003–2020) of the Household, Income and Labour Dynamics in Australia Survey. Mental health was measured using the five-item Mental Health Inventory. For each wave, we calculated population-weighted age-standardised estimates of mean Mental Health Inventory scores for people with and without disability and calculated the mean difference in Mental Health Inventory score to determine inequalities. Analyses were stratified by age, sex and disability group (sensory or speech, physical, intellectual or learning, psychological, brain injury or stroke, other).

**Results:**

From 2003 to 2020, people with disability had worse mental health than people without disability, with average Mental Health Inventory scores 9.8 to 12.1 points lower than for people without disability. For both people with and without disability, Mental Health Inventory scores decreased, indicating worsening mental health, reaching the lowest point for both groups in 2020. For some subpopulations, including young females and people with intellectual disability, brain injury or stroke, mental health inequalities worsened.

**Conclusion:**

This paper confirms that people with disability experience worse mental health than people without disability. We add to previous findings by demonstrating that disability-related inequalities in mental health have been sustained for a long period and are worsening in some subpopulations.

## Introduction

Mental health is not equally distributed in the population, with certain subgroups at higher risk of poor mental health and/or mental disorders. A key driver of this is differential exposure to disadvantaged socio-economic circumstances (Alegría et al., 2018). One group that experiences significant disadvantage is people with disability (Centre of Research Excellence in Disability and Health, 2022; Kavanagh et al., 2015b). It is, therefore, not surprising that people with disability experience worse mental health than people without disability. The considerable inequalities in mental health for people with disability are partly driven by social determinants of health such as poverty, low education, high rates of unemployment, violence and discrimination (Australian Institute of Health and Welfare, 2010, 2020; Kavanagh et al., 2012; Krahn et al., 2015; World Health Organization, 2022a) and are therefore modifiable.

A recent Australian study examining trends in mental health between 2001 and 2017 demonstrated that between 2013 and 2017, mental health declined in certain subgroups of the population, particularly the youngest and oldest (Burns et al., 2020). However, no evidence exists about whether mental health has changed for people with disability. Tracking inequalities in mental health is important, not only because they are unjust but also because, as outlined above, they are largely avoidable. Monitoring mental health inequalities helps us identify where policy effort and resources should be targeted for the largest health gain. Furthermore, Australia is a signatory to the UN Convention on the Rights of Persons with Disabilities (CRPD; United Nations, 2008; United Nations General Assembly, 2007). This legally binding treaty has committed Australia to reducing health inequalities for people with disability. However, we do not currently know if the Australian government are meeting their CRPD treaty obligations.

This study, therefore, sought to close this evidence gap by tracking disability-related inequalities in mental health over the last two decades. We analysed annual data between 2003 and 2020 from the longitudinal Household, Income and Labour Dynamics in Australia (HILDA) survey to examine population-level changes in annual mental health scores for people with and without disability.

Disability is a complex biopsychosocial phenomenon resulting from dynamic interactions between biological, personal and environmental factors, which together affect accessibility and participation within society (World Health Organization, 2002). Thus, while it is important to examine changes in mental health for people with disability compared to people without disability, because of the diverse nature of disability, there is also a need to consider changes in mental health in subgroups of people with disability. This can be done by stratifying analyses by factors such as age, sex and disability group (based on type of impairment, i.e. sensory or speech, physical, intellectual or learning, psychological, brain injury or stroke, other).

Another reason to examine mental health trends stratified by disability group is because mental conditions can be a cause of disability, leading to potential reverse causation. However, mental health represents more than the presence or absence of a mental disorder (Patel et al., 2018) and people with mental health conditions can experience high levels of mental wellbeing (World Health Organization, 2022b). This is because mental health exists on a continuum ranging from high levels of mental wellbeing to mild and time-limited distress to severely disabling conditions that are chronic and progressive, and can also fluctuate across the lifetime in response to individual, social and structural factors (Patel et al., 2018; World Health Organization, 2022b). This challenge can be addressed by examining mental health trends in disability subgroups where mental health conditions are not the cause of disability.

This study, therefore, investigated whether mental health inequalities for people with disability have changed over time by comparing the mental health of people with and without disability annually from 2003 to 2020 to investigate time-trends in disability-related mental health inequalities. To ensure that inequalities in mental health were not masked by the complexity of factors that lead to disability, we further stratified the analysis by age, sex and disability group.

## Methods

### Data source

The HILDA Survey is a longitudinal survey of Australian households that has been conducted annually since 2001 using a combination of interviews and self-completed questionnaires. Interviews are conducted on all household members who are aged 15+ years. In 2001, a national probability sample of private dwellings was made. There were 7682 responding households with 13,969 respondents, representing a household response rate of 66% (Watson, 2021). Later waves of the survey included all participants from the original sample, in addition to any children born into or adopted into the household, and new household members. In 2011, 2153 households and 4009 respondents were added to maintain representativeness. This top-up sample had a household response rate of 69% (Watson, 2021). Over the first 20 waves, 33,347 participants have been interviewed. Individual response rates for continuing participants was approximately 95% across the waves (Summerfield et al., 2021). Further details are available elsewhere (Summerfield et al., 2021; Watson and Wooden, 2012).

Ethical approval for the HILDA Survey was granted by the Human Research Ethics Committee of the University of Melbourne in 2001 (ID 1955879) and has been updated or renewed annually. Consent for participation in the HILDA Survey is obtained via use of an information letter to potential respondents, which details that informed consent is implied when participants agree to be interviewed. Parents/guardians provide consent for children aged 15–17 years.

### Disability measures

In every wave of the HILDA Survey, participants were asked ‘do you have any long-term health condition, impairment or disability (such as these) that restricts you in your everyday activities, and has lasted or is likely to last, for 6 months or more?’ To help, all respondents were shown flashcards with example conditions, impairments or disabilities that align with the International Classification of Functioning, Disability and Health (ICF) framework (World Health Organization, 2002). In 2003, the flashcards were updated to include a broader range of conditions. Thus, to ensure consistency in the definition of disability over time, we use data from 2003 onwards.

We used the responses to these flashcard items to classify participants who had a disability into six disability groups: sensory and speech (sight problems not corrected by glasses; hearing problems; speech problems), physical (including blackouts; difficulty gripping things; limited use of legs or feet and restricted activities due to chronic pain), intellectual and learning (difficulty learning or understanding things), psychological (mental illness; nervous or emotional condition), brain injury and stroke (long-term effects resulting from head injury, stroke or other brain damage), other/type not specified (other long-term conditions that restricted everyday activities or impairment types, which were not otherwise specified). These disability groups are based on those used by the 2018 Survey of Disability, Ageing and Carers (SDAC; Australian Bureau of Statistics, 2019a), the most comprehensive source of data on disability prevalence in Australia. However, due to the phrasing of disability questions within the HILDA Survey, we were unable to match the SDAC group for ‘psychosocial’; we therefore used the disability group definition for ‘psychological’, which is consistent with the SDAC grouping prior to 2015 but slightly narrower than the disability group termed ‘psychosocial’ used in SDAC from 2015 onwards (see Supplementary Information; Australian Bureau of Statistics, 2013). Note that people with disability can belong to more than one disability group, thus the sum of prevalences in the subgroups exceeds 100%.

### Mental health measure

The five-item Mental Health Inventory (MHI), a subscale of the 36-item Short-Form Health Survey (SF-36), was used to assess mental health. The MHI assesses symptoms of depression and anxiety, in addition to positive aspects of mental health, in the previous 4 weeks. The scale was constructed from the five items that best predicted the summary score of the more comprehensive 38-item Mental Health Inventory used in the Medical Outcomes Study questionnaire and is an effective screening instrument for mood disorders or severe depressive symptomatology (Rumpf et al., 2001; Yamazaki et al., 2005). The MHI, via the SF-36, was collected during all waves of the HILDA Survey. It is a continuous measure ranging from 0 to 100, where higher scores indicate better mental health. A difference of 4–5 points on the MHI scale represents a clinically important difference in mental health symptoms (Knox and King, 2009; McHorney et al., 1993).

### Age standardisation

Age is related to both disability (Australian Institute of Health and Welfare, 2020) and mental health (Burns et al., 2020), thus is likely to be an important confounder of differences in mental health between people with and without disability. To adjust for this confounding, we used direct age standardisation. We chose to standardise to the age distribution of people with disability in 2020 (the most recent wave) of the HILDA Survey, using 5-year age groups that were top-coded at 85+ years. This means our estimates of mental health for people with disability are largely unadjusted by the standardisation procedure, while estimates for people without disability are adjusted and provide mental health scores as though they had the same age profile as the 2020 disabled population. This approach to standardisation supports self-determination for the population of interest because the disability group mental health estimates reflect their reality rather than being weighted to the general population age distribution, which can be substantially different (Robson et al., 2007; Thurber et al., 2022).

### Statistical analysis

The analytic sample consisted of 278,057 observations, obtained from 31,040 participants aged 15+ years between 2003 and 2020, who responded to the disability question (99.9% of survey respondents). Population-weighted descriptive analyses were conducted to describe the Australian population aged 15+ years with and without disability, in terms of the distribution of age (based on participant age on 30 June for each wave) and sex, for the first and last wave contributing to the analysis (2003 and 2020). We did this using survey weights provided by HILDA to adjust for clustering and stratification in the survey design and non-response, employing the Taylor Series linearisation method for standard error calculation (Hayes, 2008). Disability group was also analysed for participants with a disability.

To compare the mental health of people with and without disability, we calculated population-weighted age-standardised estimates of the mean MHI scores for each wave of the HILDA Survey. We then calculated the mean difference in MHI scores between people with and without disability for each wave. Further analysis was performed for subpopulations by sex and/or 10-year age groups, and by disability group.

To assess potential confounding of mental health inequalities by the presence of psychological disability, which can be a cause of disability in some individuals, we separated people with disability into two groups: (1) people with disability who have a psychological disability and (2) people with disability who did not have a psychological disability. Our sensitivity analysis first compared mean MHI scores for people with disability who did not have psychological disability to people without disability. We then compared the mean MHI scores for people with disability who have psychological disability to people with disability who do not have psychological disability.

All statistical analyses were performed using Stata SE version 17.0. Graphs were prepared using R version 4.2.1, RStudio version 2022.07.1 and ggplot2 version 3.3.6.

### Missing data

There were missing data for MHI scores for 10.7% of observations. Analysis of the missing MHI data, when stratified by disability, demographic and socio-economic characteristics suggested that data were not missing completely at random (see Supplementary Information), but could be missing at random (i.e. the probability of missingness was conditional on the observed data but not the missing data). Based on the framework of Lee and colleagues for the handling of missing data in observational studies (Lee et al., 2021), given that the probability of missingness of the outcome measure is expected to depend on the outcome or exposure, a complete case analysis was likely to be biased. To reduce potential bias, multiple imputation for MHI scores using predicted mean matching with 50 imputations was performed (see Supplementary Information for further details). To account for the possibility that data may have been missing not at random (i.e. missingness depended on the value even within strata, such that missing MHI scores were dependent on an individual’s mental health status), a sensitivity analysis was conducted using complete case analysis and we observed little difference in results to those from the imputed data (data not shown).

## Results

### Population characteristics

Table 1 shows population-weighted demographic characteristics, stratified by disability, for the first and final waves of data. The prevalence of disability was 27.8% (95% confidence interval [CI] = [26.6%, 29.1%]) in 2003 and 29.3% (95% CI = [28.0%, 30.6%]) in 2020. People with disability were older than people without disability. The mean age of people with disability was 53.6 years (95% CI = [52.6, 54.6]) in 2003 and 55.1 years (95% CI = [54.2, 56.0]) in 2020, while the mean age of people without disability was 39.6 years (95% CI = [39.1, 40.2]) in 2003 and 41.6 years in 2020 (95% CI = [41.1, 42.1]). There were almost equal proportions of males and females within the population, including when stratified by disability.

**Table 1.** Population-weighted estimates of demographic characteristics, and disability groups, of people with and without disability in 2003 and 2020.

Characteristic	2003		2003		2020		2020	
	No disability		Any disability		No disability		Any disability	
	(*n* = 9175)		(*n* = 3553)		(*n* = 11,953)		(*n* = 5112)	
	%	95% CI	%	95% CI	%	95% CI	%	95% CI
Age (years)								
15–24	21.5	[20.2, 22.9]	7.8	[6.8, 9.0]	18.0	[16.9, 19.1]	9.6	[8.3, 11.0]
25–34	21.1	[19.8, 22.5]	10.3	[9.1, 11.7]	21.6	[19.5, 23.8]	10.4	[9.4, 11.6]
35–44	21.0	[19.9, 22.1]	14.0	[12.8, 15.4]	19.9	[18.3, 21.5]	9.1	[8.1, 10.2]
45–54	17.5	[16.5, 18.6]	17.2	[15.6, 18.9]	16.0	[14.9, 17.2]	15.1	[13.4, 17.0]
55–64	10.0	[9.2, 10.9]	19.5	[17.8, 21.3]	12.7	[11.8, 13.3]	18.7	[17.3, 20.1]
65–74	6.0	[5.3, 6.8]	15.3	[13.8, 16.9]	8.1	[7.3, 9.0]	19.5	[17.9, 21.1]
75+	2.8	[2.4, 3.3]	15.9	[13.9, 18.0]	3.8	[3.3, 4.3]	17.6	[16.1, 19.3]
Sex								
Male	48.9	[47.8, 50.0]	50.1	[48.4, 51.9]	49.8	[48.6, 51.1]	47.2	[45.5, 48.8]
Female	51.1	[50.0, 52.2]	49.9	[48.1, 51.6]	50.2	[48.9, 51.4]	52.8	[51.2, 54.5]
Disability group^a^								
Sensory and speech			24.1	[22.5, 25.7]			21.8	[20.2, 23.5]
Physical			56.8	[54.7, 58.9]			60.7	[58.6, 62.9]
Intellectual and learning			3.1	[2.4, 4.1]			6.8	[5.8, 8.0]
Psychological			10.8	[9.5, 12.2]			21.4	[19.9, 23.1]
Brain injury and stroke			3.6	[2.9, 4.6]			4.7	[3.9, 5.6]
Other/type not specified			55.9	[53.8, 58.0]			59.1	[57.1, 61.0]

CI: confidence interval.

a Only available for people with disability. Note that people may have multiple disabilities and belong to more than one disability group, thus the percentages do not tally to 100.

Within the subpopulation of people who had a disability, in 2020 the most common disability group was physical (60.7%), followed by sensory or speech (21.8%) and psychological (21.4%). About 6.8% of people with disability had an intellectual or learning disability and 4.7% had a brain injury or stroke, while 59.1% had another type of disability. While the prevalence of disability within the population changed little between 2003 and 2020, the proportions within disability groups did change. This was most noticeable for psychological disability, which almost doubled from 10.8% in 2003 to 21.4% in 2020. Furthermore, the proportion of people with an intellectual or learning disability more than doubled from 3.1% in 2003 to 6.8% in 2020.

### Mental health inequalities by disability

**Figure 1.**
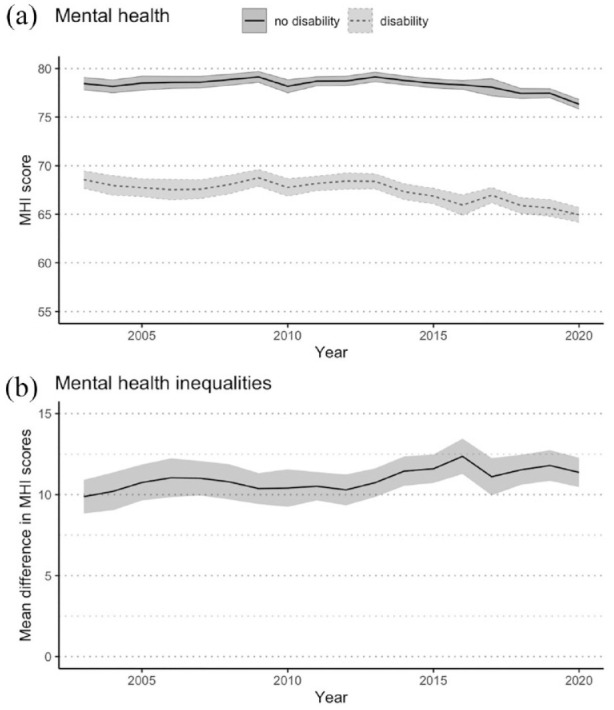
Mental health over time for Australians aged 15 + years with and without disability, 2003–2020. (A) Mental health: age-standardised population-weighted mean MHI scores over time, with 95% confidence intervals. (B) Mental health inequalities: mean difference in MHI scores over time, with 95% confidence intervals.

Figure 1(A) shows that across all 18 waves, people with disability have a lower MHI score and therefore poorer mental health than people without disability. However, MHI scores decreased for both people with and without disability across the 18 waves, reaching the lowest point in 2020 where people with disability had a mean MHI score of 64.9 (95% CI = [64.2, 65.7]), while people without disability had a mean MHI score of 76.3 (95% CI = [75.8, 76.8]). Figure 1(B) shows that the mean difference in MHI scores, comparing people with and without disability, increased from 9.9 (95% CI = [8.8, 10.9]) in 2003 to 11.4 (95% CI = [10.5, 12.3]) in 2020. Across the 18 years examined, the greatest inequality was observed in 2016 with a difference of 12.4 (95% CI = [11.3, 13.5]), while the smallest inequality was observed in 2003.

### Mental health inequalities by disability, age and sex

**Figure 2.**
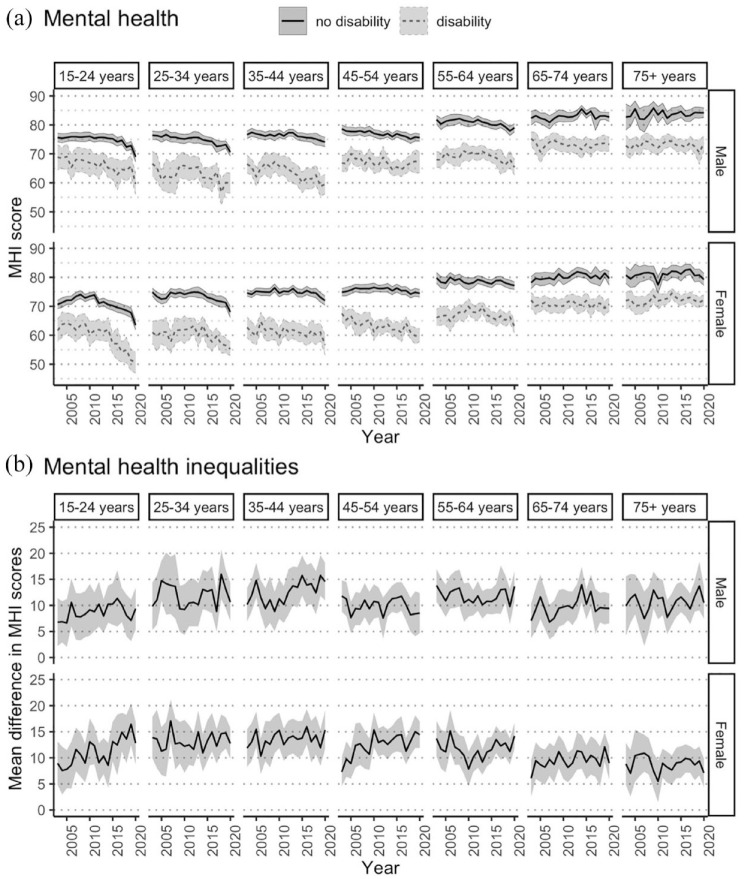
Mental health over time for Australians aged 15+ years with and without disability, 2003–2020, stratified by age group and sex. (A) Mental health: age-standardised population-weighted mean MHI scores over time, stratified by age group and sex, with 95% confidence intervals. (B) Mental health inequalities: mean difference in MHI scores over time, stratified by age group and sex, with 95% confidence intervals.

Figure 2(A) demonstrates that when stratified by both age and sex, there are mental health inequalities for people with disability in all subgroups. Mental health is better in older age groups, with higher MHI scores for older people regardless of disability. When considering trends over time, MHI scores decreased for people aged < 65 years, regardless of disability, but were relatively stable over the 18-year period for people with and without disability aged 65+ years. Figure 2(B) shows that disability-related mental health inequalities were persistent over time and appeared to widen for females aged 15–24 years and females aged 45–54 years, with the inequalities being more pronounced than for males of the same age.

In contrast, when stratified only by age or by sex, persistent inequalities in MHI scores were observed between people with and without disability in all subgroups; however, some of the disability-related inequalities observed in Figure 2 for certain subgroups were masked when the interaction effect of age and sex was not considered (see Supplementary Information).

### Mental health inequalities by disability group

**Figure 3.**
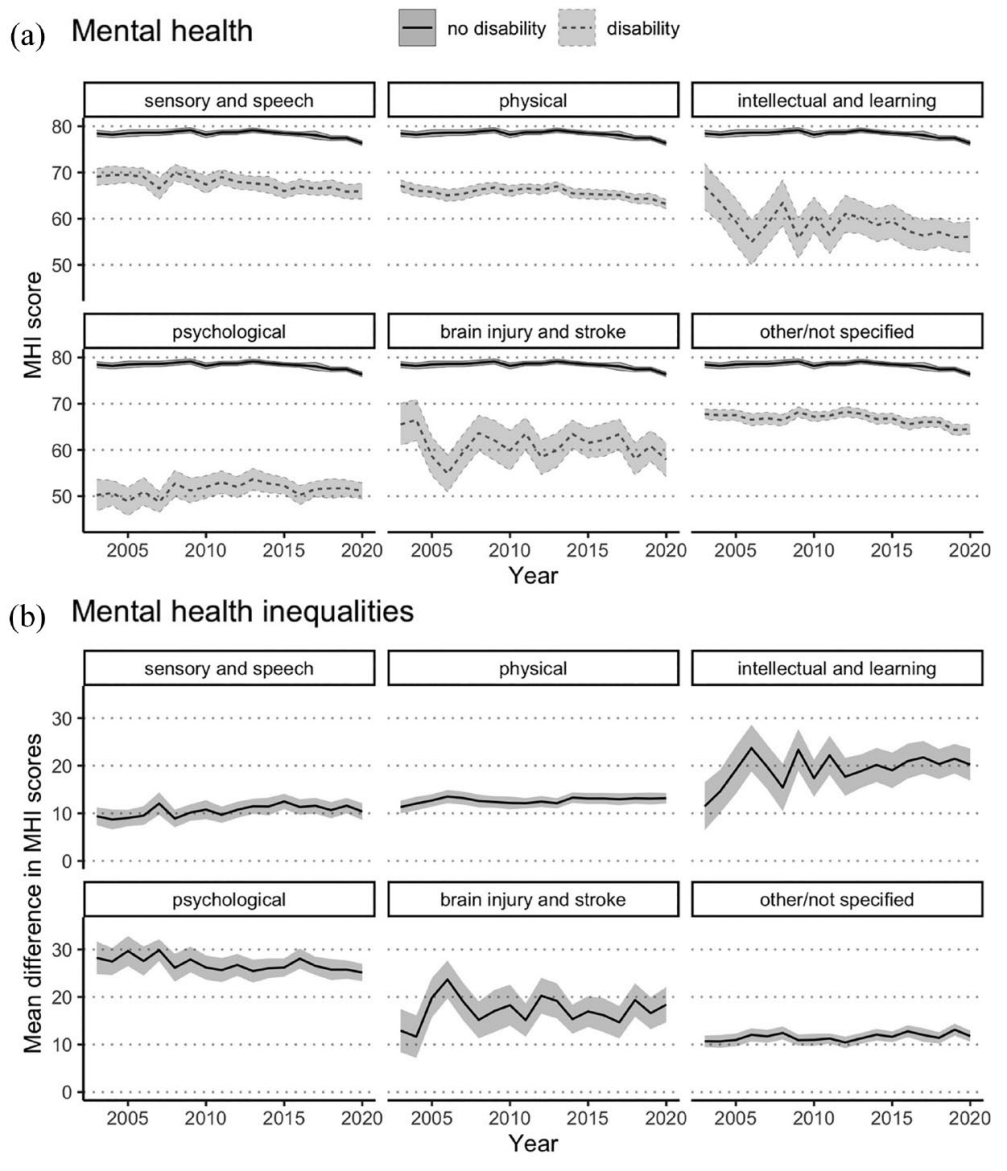
Mental health over time for Australians aged 15+ years with and without disability, 2003–2020, stratified by disability group. (A) Mental health: age-standardised population-weighted mean MHI scores over time, stratified by disability group, with 95% confidence intervals. (B) Mental health inequalities: mean difference in MHI scores, stratified by disability group, with 95% confidence intervals. Note that individuals with disability may belong to more than one disability group.

Figure 3(A) shows that mean MHI scores were consistently lower for people with disability than for people without disability, regardless of disability group. People with intellectual or learning disability, psychological disability, and/or brain injury or stroke had lower MHI scores on average than people with sensory or speech disabilities, physical disability, or other types of disability.

Figure 3(B) demonstrates that the greatest inequality in mean MHI scores between disability group and the non-disabled population was observed for people with psychological disability. Inequalities increased across the 18 years for people with intellectual or learning disability, with the mean difference in 2020 (20.2; 95% CI = [16.8, 23.6]) being almost double that observed in 2003 (11.5; 95% CI = [6.4, 16.5]).

### Sensitivity analysis

People with psychological disability had, on average, consistently low MHI scores across the 18 years that on average were 26.9 points lower than people without disability. People with non-psychological disability had MHI scores that on average across the 18 years were 7.9 points lower than people without disability. Further details are included in the Supplementary Information.

## Discussion

This study found persistent mental health inequalities between people with and without disability between 2003 and 2020 in Australia. For some subpopulations, such as young women and people with intellectual disability, brain injury or stroke, mental health inequalities between people with and without disability widened.

For every year during the 18-year period from 2003 to 2020, people with disability had worse mental health than people without disability, with average MHI scores being 9.8 to 12.1 points lower than for people without disability. Given that a change in MHI scores of 4–5 points is clinically meaningful (Knox and King, 2009; McHorney et al., 1993), this finding represents a substantial and clinically relevant difference in mental health between people with and without disability. Our finding reinforces other studies that have reported mental health inequalities for people with disability (including acquired disability; Aitken et al., 2017, 2018, 2020; Kavanagh et al., 2015a, 2016; Lucas, 2007; Mandemakers and Monden, 2010; Smith et al., 2005; Yang and George, 2005), but adds to previous findings by demonstrating that the inequalities are not mere snapshots in time, but have been sustained for decades and are not improving.

It is noteworthy that mental health inequalities for people with disability did not improve over the 18 years, irrespective of sex or age. Moreover, we observed a trend towards worsening mental health scores and increasing mental health inequalities in younger females with disability. This evidence of increasing mental health inequalities for young people with disability is concerning given that it occurs at an important life transition point, indicating a need for policy solutions that enable healthy adult transitions for young people with disability.

While the type of disability affected average MHI scores, people from all disability groups had substantial mental health inequalities compared to people without disability. The largest inequality was observed for people with psychological disability, which was not unexpected given that ‘psychological disability’ is defined as having a mental illness or a nervous or emotional condition (Australian Bureau of Statistics, 2013). It is also noteworthy that the proportion of people with disability who reported a psychological disability doubled between 2003 and 2020. Moreover, we observed that people with intellectual or learning disability and/or brain injury or stroke not only had substantial mental health inequalities, but showed evidence of widening inequalities over time. Our findings are consistent with findings from the National Health Survey in 2017–2018 that people in these disability groups were more likely to experience high or very high levels of psychological distress than people without disability (Australian Institute of Health and Welfare, 2020). Thus, targeting of services for disability groups may be beneficial for reducing mental health inequalities.

While the present time series analysis did not examine the effects of specific drivers on the observed mental health inequalities, demographic and socio-economic characteristics are likely to play a role given that mental health inequalities for people with disability are at least partly driven by social determinants of health (Australian Institute of Health and Welfare, 2010; Kavanagh et al., 2012; Krahn et al., 2015; World Health Organization, 2022a). For instance, our previous research estimated that 39% of the mental health inequalities observed in Australian working-aged adults with acquired disability could be explained by material socio-economic factors including employment, income and financial hardship (Aitken et al., 2018). Among adults with disability, other factors such as living in unaffordable housing (Kavanagh et al., 2016), having lower wealth (Kavanagh et al., 2015a), being in the lowest income quintile or not being in a relationship (Aitken et al., 2017), and being in low skilled employment (Aitken et al., 2020) prior to disability acquisition are all associated with poorer mental health. Consideration is also needed regarding the barriers to participation that people with disability face in employment, economic life, transport, community, leisure and civic activities, social contact and accessibility, which we recently demonstrated mediate inequalities in mental health and wellbeing in people with acquired disability (Aitken et al., 2022).

This study has several strengths. We used a large, nationally representative sample covering 2003 to 2020. The age distribution of people with and without disability who were interviewed was fairly consistent with that in the Australian population aged 15+ years, as determined by the SDAC (Australian Bureau of Statistics, 2019b). To address differences in age distribution in people with and without disability, we employed age-standardisation based on a reference population of people with disability, which ensures that the results reported reflect the reality of our population of interest (Robson et al., 2007; Thurber et al., 2022).

There were also limitations. The prevalence of disability within the sample was higher than reported in Australian national statistics (Australian Institute of Health and Welfare, 2020), which may relate to slightly different definitions of disability. The HILDA Survey is focussed on people living within the community, thus people with severe disability, who are more likely to reside in care homes and institutions, are underrepresented in the sample. This may lead to an underestimate in mental health inequalities because people with more severe disability tend to report poorer mental health (Australian Institute of Health and Welfare, 2020). Our disability sample included people with psychological disability, whom we would expect to have poorer mental health. In our sensitivity analysis excluding people with psychological disability, there was evidence of attenuated, but still clinically meaningful, differences in MHI scores of 7.9 points on average across the 18 years between people with non-psychological disability and people without disability. There may also be selection bias due to missing data, however multiple imputation of the outcome measure using six key demographic and socio-economic variables that had complete data would have decreased potential selection bias. Measurement error may have occurred since all variables were self-reported, which may have introduced bias, particularly if reporting of mental health was systematically different between people with and without disability.

Given that poor mental health results in considerable costs to individuals, their families and carers, and to society (World Health Organization, 2022b), addressing mental health inequalities is essential. Yet despite considerable commitment and investment by the Australian Government, it is evident from this study that mental health inequalities for people with disability are substantial, persistent and have not improved between 2003 and 2020. This includes commitment to the CRPD (United Nations, 2008), implementation of the 2010–2020 National Disability Strategy in which mental health was recognised as a policy priority (Council of Australian Governments, 2011), and the progressive implementation, from 2016, of the National Disability Insurance Scheme (NDIS) to improve societal participation and quality of life for people with severe permanent disability (Buckmaster and Clark, 2018). The recent release of Australia’s Disability Strategy 2021–2031 (Department of Social Services, 2021) recognised mental health inequalities are an ongoing problem and that mental health supports that are appropriate and accessible for people with disability are needed. However, until the causes of these persistent and widening inequalities are understood and addressed, it will be challenging to develop policies that effectively reduce mental health inequalities for people with disability. Indeed, given the complex interplay of the social determinants of health in both disability and mental health, along with greater and persistent socio-economic disadvantage experienced by people with disability (Centre of Research Excellence in Disability and Health, 2022; Kavanagh et al., 2015b), addressing mental health inequalities for people with disability requires considerable change in social structure and policy.

## Supplemental Material

sj-docx-1-anp-10.1177_00048674231193881 – Supplemental material for Trends in mental health inequalities for people with disability, Australia 2003 to 2020 Click here for additional data file. Supplemental material, sj-docx-1-anp-10.1177_00048674231193881 for Trends in mental health inequalities for people with disability, Australia 2003 to 2020 by Glenda M Bishop, Anne Marie Kavanagh, George Disney and Zoe Aitken in Australian & New Zealand Journal of Psychiatry
