# Supplementary material for: Trends in mental health inequalities for people with disability, Australia 2003 to 2020
Source: Aust N Z J Psychiatry. 2023 Aug 22;57(12):1570–9. doi: 10.1177/00048674231193881 (PMC10666511; doi:10.1177/00048674231193881)
Supplement: sj-docx-1-anp-10.1177_00048674231193881 – Supplemental material for Trends in mental health inequalities for people with disability, Australia 2003 to 2020 [file sj-docx-1-anp-10.1177_00048674231193881.docx]

**Supplementary Information**

Trends in mental health inequalities for people with disability, Australia 2003 to 2020.

Bishop GM, Kavanagh AM, Disney G, Aitken Z.

**Methods**

*Alignment of disability groups between HILDA and SDAC*

We classified participants with disability into the six disability groups by matching disability-related questions asked within the HILDA Survey to those asked within the SDAC. Supplementary Table 1 demonstrates how the HILDA variables (as coded within the dataset) map to the SDAC 2018 Household Survey Questionnaire modules (Australian Bureau of Statistics, 2018) and how they were classified into the six disability groups (Australian Bureau of Statistics, 2019a).

Prior to 2015, the SDAC included a disability group termed ‘psychological’ (Australian Bureau of Statistics, 2013), which is consistent with the term (and disability group) used in the present study. However, in 2015 the SDAC introduced the ‘psychosocial’ disability group, which is an expansion of the ‘psychological’ disability group to also include ‘memory problems or periods of confusion’ and ‘social or behavioural difficulties’ that restrict everyday activities (Australian Bureau of Statistics, 2019a). Given that it is not possible to match the HILDA Survey variables to the SDAC ‘psychosocial’ disability group, and that three-quarters of the data used in the present study was obtained prior to the SDAC classification change, we have used the ‘psychological’ disability group in this study.

*Analysis of missing data*

Of the 278,057 observations obtained during 2003 to 2020 from people aged 15 years and over who responded to the disability question, 10.7% (n = 29,832) had missing values for MHI scores. Across the 18 waves, the proportion of observations with missing values for MHI scores varied from 8.3% to 14.0%. Stratification of missing MHI scores by disability, demographic and socio-economic characteristics can be seen in Table 2. Participants with missing MHI scores were more likely to be under 35 years or 75 years and above, male and unemployed, but were less likely to have a Bachelor’s degree or higher education. Furthermore, MHI scores were more likely to be missing for participants who had intellectual or learning disabilities, or who had experienced a brain injury or stroke. However, it should be noted that 94.9% of the observations with missing values for the MHI scores occurred because participants did not return the Self-Completion Questionnaire that contained the questions for the SF-36 from which the MHI scores were derived. The remaining 5.1% of observations with missing MHI values resulted from participants either refusing to answer or providing invalid responses for more than 2 of the 5 questions in the MHI subscale, given that MHI scores can only be derived if 3 or more questions are answered.

*Multiple imputation*

The imputation model included the variables listed in Supplementary Table 2 using predictive mean matching based on the 5 nearest neighbours (closest observations), with the observations subsampled by survey wave. This ensured that the values for MHI scores were limited to the observed range of the scale (i.e. between 0 and 100), while preserving the non-normal distribution of the observed values. Imputation using subsamples for each wave of the survey accommodated for potential effects that may have been caused by changes to mental health (and its determinants) over the 18 years of data collection. The population-weighted estimates for each wave of the HILDA Survey were determined for each of the 50 imputed datasets, from which an overall estimate of the mean MHI scores were obtained. Standard errors were derived using Rubin rules for combining the between-imputation and within-imputation variance, obtained from the Taylor Series linearisation standard errors of the mean MHI estimates.

**Results**

*Age distribution*

Given the considerable difference in the population-weighted proportions between age groups of people with and without disability (shown in Table 1 of the manuscript), further analysis of the age distributions of these two subpopulations was undertaken. The population pyramids for unweighted sample proportions for 2003 and 2020 (Supplementary Figure 1A and 1B, respectively) demonstrate a mostly consistent pattern over time such that people with disability were more likely to be older than people without disability; this pattern was similar across all 18 waves of the HILDA Survey (data not shown). Furthermore, except for people aged 80 years and above, the age distribution of people with and without disability within the analytic sample was consistent with that observed in the Australian population aged 15 years and over, as determined by the 2018 SDAC (Supplementary Figure 1C).

*Mental health inequalities by disability and age*

Supplementary Figure 2A shows that there are mental health inequalities for people with disability in all age groups. Higher MHI scores were observed for older people whether they have a disability or not, and remained relatively stable over the 18-year period. However, regardless of disability, MHI scores decreased for people aged under 65 years over the 18-year period. Supplementary Figure 2B shows that disability-related inequality in mental health did not decrease for any age group and appeared to increase over time for the youngest age group (15-24 years).

*Mental health inequalities by disability and sex*

Supplementary Figure 3A shows that there are mental health inequalities for people with disability for both sexes. There was however little variation between males and females with regards to the disability-related inequality in mental health (Supplementary Figure 3B).

*Sensitivity analysis for impact of psychological disability on MHI scores*

Given that psychological disability includes mental illness or nervous or emotional conditions, which will impact on MHI scores, we conducted a sensitivity analysis to determine whether psychological disability was underlying the difference in MHI scores between people with and without disability. Supplementary Figure 4A shows that the age-standardised population-weighted mean MHI scores were considerably lower for people with psychological disability when compared to people with disability but without psychological disability, and when compared to people without disability. In 2020, people with disability but without psychological disability had a mean MHI score of 68.7 (95% CI: 67.9, 69.5), which had decreased compared to the mean MHI score in 2003 (70.7; 95% CI: 69.9, 71.6), consistent to the pattern observed for people without disability. This is in contrast to the mean MHI scores for people with psychological disability, which were largely unchanged over the 18 years examined. Supplementary Figure 4B shows that the mental health inequalities for people with disability but without psychological disability, when compared to people without disability, were largely unchanged over the 18 years; however, the size of the inequality was approximately 3 points smaller when compared to that for the whole disability sample versus people without disability.

**References**

Australian Bureau of Statistics (2013) *4430.0 - Disability, Ageing and Carers, Australia: Summary of Findings, 2012. Appendix 2 - Disability Groups*. Available at: <https://www.abs.gov.au/ausstats/abs@.nsf/Previousproducts/4430.0Appendix902012?opendocument&tabname=Notes&prodno=4430.0&issue=2012&num=&view>= (accessed 24 Apr).

Australian Bureau of Statistics (2018) *Survey of Disability, Ageing and Carers (SDAC 18) - Household Survey Questionnaire [Excel file]*. Available at: <https://www.abs.gov.au/statistics/health/disability/disability-ageing-and-carers-australia-summary-findings/2018/4430.0%20-%20Household%20Questionnaire.xlsx> (accessed 1 Aug).

Australian Bureau of Statistics (2019a) *Disability, Ageing and Carers, Australia: Summary of Findings methodology. Appendix - disability groups*. Available at: <https://www.abs.gov.au/methodologies/disability-ageing-and-carers-australia-summary-findings/2018#appendix-disability-groups> (accessed 24 Apr).

Australian Bureau of Statistics (2019b) *Disability, Ageing and Carers, Australia. 2018 Disability Tables [Dataset]*. Available at: <https://www.abs.gov.au/statistics/health/disability/disability-ageing-and-carers-australia-summary-findings/latest-release#data-download> (accessed 4 Apr).

**Supplementary Table 1.** Mapping of HILDA Survey variables to SDAC disability groups and the SDAC Household Survey Questionnaire modules.

| SDAC disability group | HILDA question | HILDA variable | SDAC Module ^a^ |
| --- | --- | --- | --- |
| Sensory or speech | Sight problems not corrected by glasses/lenses | hespnc | 2.1 |
|  | Hearing problems | hehear | 2.2 |
|  | Speech problems | hespch | 2.3 |
| Physical | Blackouts, fits or loss of consciousness | hebflc | 2.6 |
|  | Limited use of arms or fingers | heluaf | 2.8 |
|  | Difficulty gripping things | hedgt | 2.9 |
|  | Limited use of feet or legs | helufl | 2.10 |
|  | Any condition that restricts physical activity or physical work (e.g. back problems, migraines) | hecrpa | 2.12 |
|  | Any disfigurement or deformity | hedisf | 2.13 |
|  | Shortness of breath or difficulty breathing | hesbdb | 2.4 |
|  | Chronic or recurring pain | hecrp | 2.5 |
| Intellectual or learning | Difficulty learning or understanding things | heslu | 2.7 |
| Psychological | A nervous or emotional condition which requires treatment | henec | 2.11 |
|  | Any mental illness which requires help or supervision | hemirh | 2.14 |
| Brain injury or stroke | Long term effects as a result of a head injury, stroke or other brain damage | hehibd | 2.17 |
| Other | Long-term condition or ailment which is still restrictive even though it is being treated | hemed | 2.18 |
|  | Any other long-term condition such as arthritis, asthma, heart disease, Alzheimer’s, dementia, etc | heoth | 2.19 & 2.20 |
|  | Refused to answer | herf |  |
|  | Don’t know | hedk |  |

*^a^ SDAC Module relates to the module within the SDAC 2018 Household Survey Questionnaire that asks disability-specific questions (Australian Bureau of Statistics, 2018). Note that SDAC Modules 2.15 (memory problems or periods of confusion) and 2.16 (social or behavioural difficulties), which were added to SDAC in 2015 to extend the ‘psychological’ disability group into the ‘psychosocial’ disability group, do not map to any HILDA Survey questions associated with disability or impairment type.*

**Supplementary Table 2.** Observations that had missing values for MHI scores, stratified by disability, demographic and socio-economic characteristics.

|  | Number of observations with missing values for MHI scores | % of observations with missing values |
| --- | --- | --- |
| Analytic sample ^a^ | 29,832 | 10.7% |
| Disability |  |  |
| No disability | 21,210 | 10.6% |
| Any disability | 8,652 | 11.0% |
| Age (years) |  |  |
| 15-24 | 7,208 | 14.6% |
| 25-34 | 7,016 | 14.3% |
| 35-44 | 4,952 | 10.6% |
| 45-54 | 3,924 | 8.5% |
| 55-64 | 2,524 | 6.5% |
| 65-74 | 1,643 | 5.9% |
| 75+ | 2,595 | 12.7% |
| Sex |  |  |
| Male | 15,551 | 11.8% |
| Female | 14,311 | 9.8% |
| Education |  |  |
| Bachelor’s degree or higher | 5,100 | 7.8% |
| Year 12, Advanced Diploma, Diploma, Certificate | 13,775 | 10.9% |
| Year 11 or below | 10,987 | 12.8% |
| Employment |  |  |
| Employed | 18,808 | 10.7% |
| Unemployed | 1,627 | 15.0% |
| Not in labour force | 9,427 | 10.4% |
| Disability group ^b^ |  |  |
| Sensory and speech | 2,317 | 12.1% |
| Physical | 5,160 | 10.8% |
| Intellectual and learning | 896 | 21.9% |
| Psychological | 1,745 | 12.7% |
| Brain injury and stroke | 515 | 16.7% |
| Other/type not specified | 4,958 | 10.9% |

*^a^ Includes people aged 15 years and over, who responded to the disability question, who were interviewed during in waves 3 to 20 (2003 to 2020).*

*^b^ Only available for people with disability, who may be included in more than one disability group.*

**
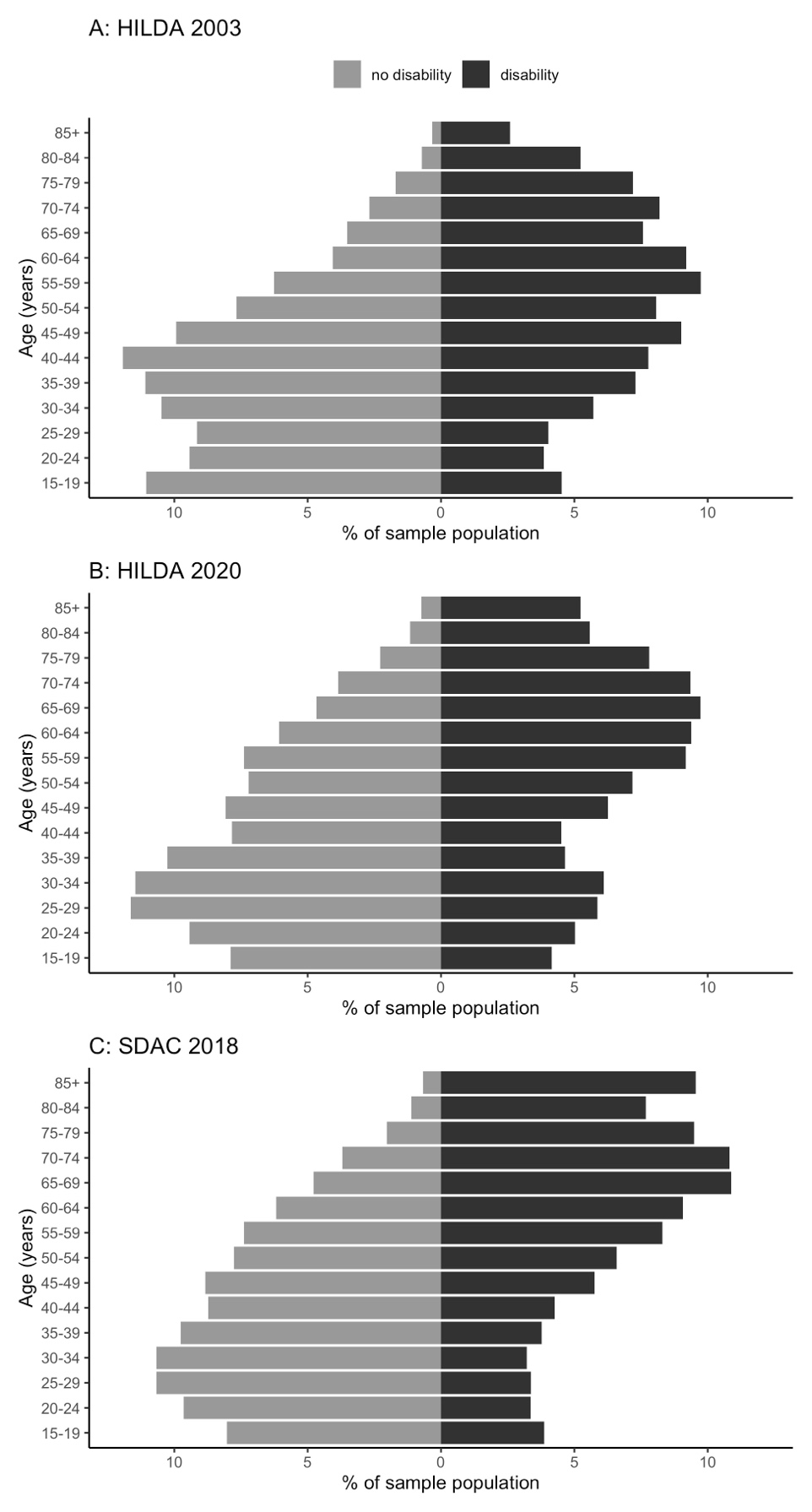
**

**Supplementary Figure 1.** Population pyramids comparing the age distributions for people with and without disability, aged 15+ years. A: HILDA sample population in 2003 (wave 3). B: HILDA sample population in 2020 (wave 20). C: Australian population obtained from the 2018 Survey of Disability, Ageing and Carers (Australian Bureau of Statistics, 2019b).


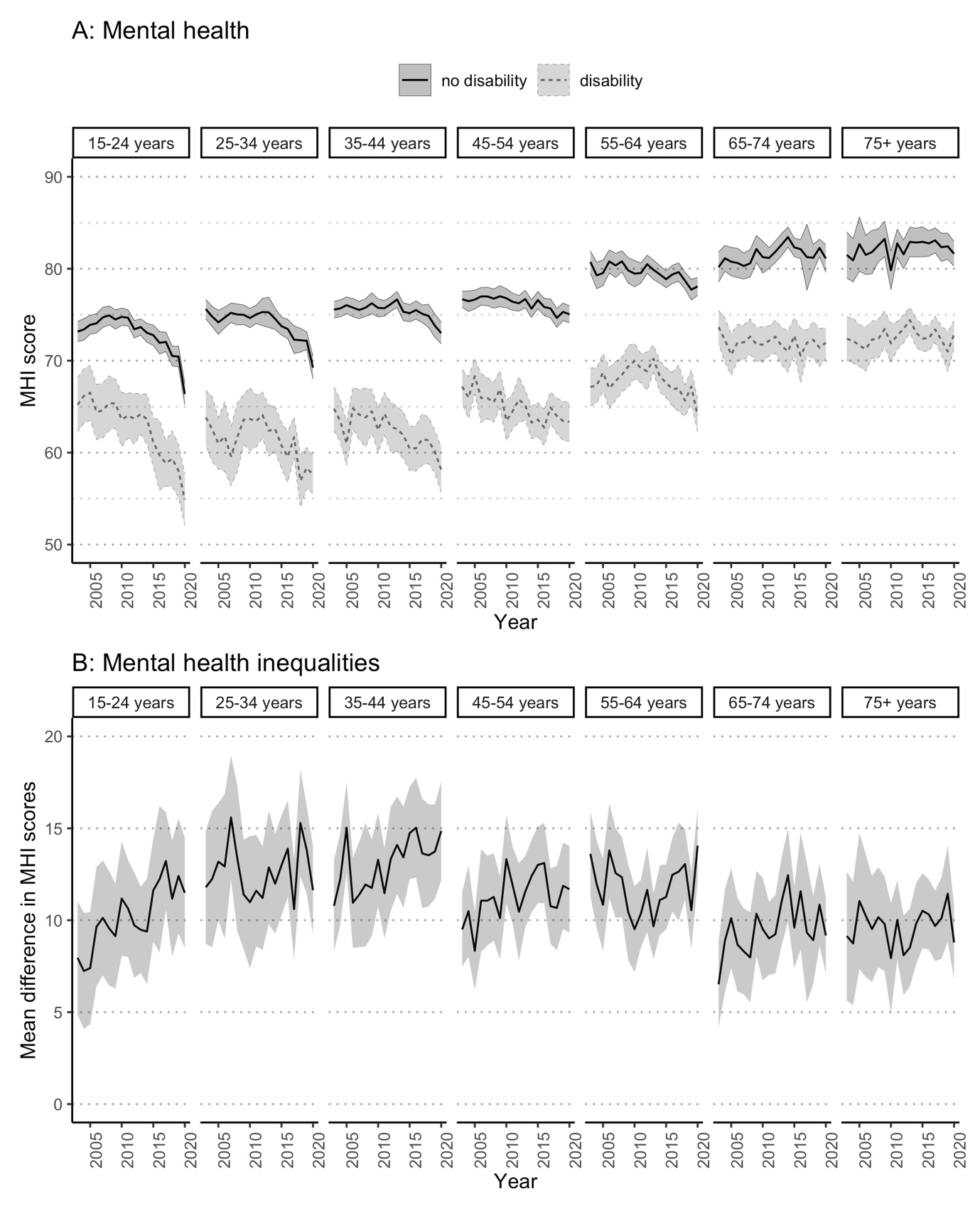


**Supplementary Figure 2.** Mental health over time for Australians aged 15+ years with and without disability, 2003-2020, stratified by age group. A: Age-standardised population-weighted mean MHI scores over time, stratified by age group, with 95% confidence intervals. B: Mean difference in MHI scores over time, stratified by age group, with 95% confidence intervals.


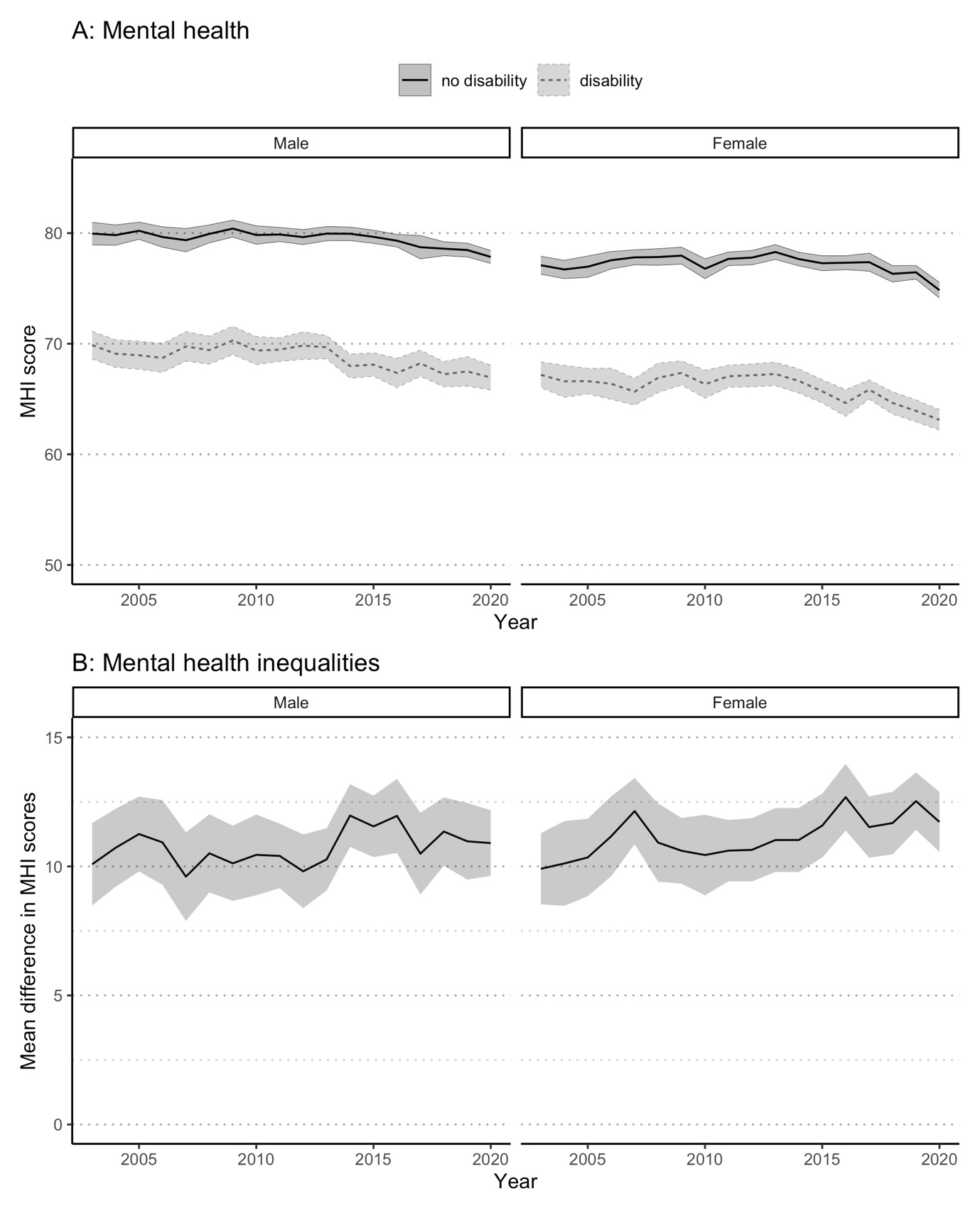


**Supplementary Figure 3.** Mental health over time for Australians aged 15+ years with and without disability, 2003-2020, stratified by sex. A: Age-standardised population-weighted mean MHI scores over time, with 95% confidence intervals. B: Mean difference in MHI scores over time, with 95% confidence intervals.


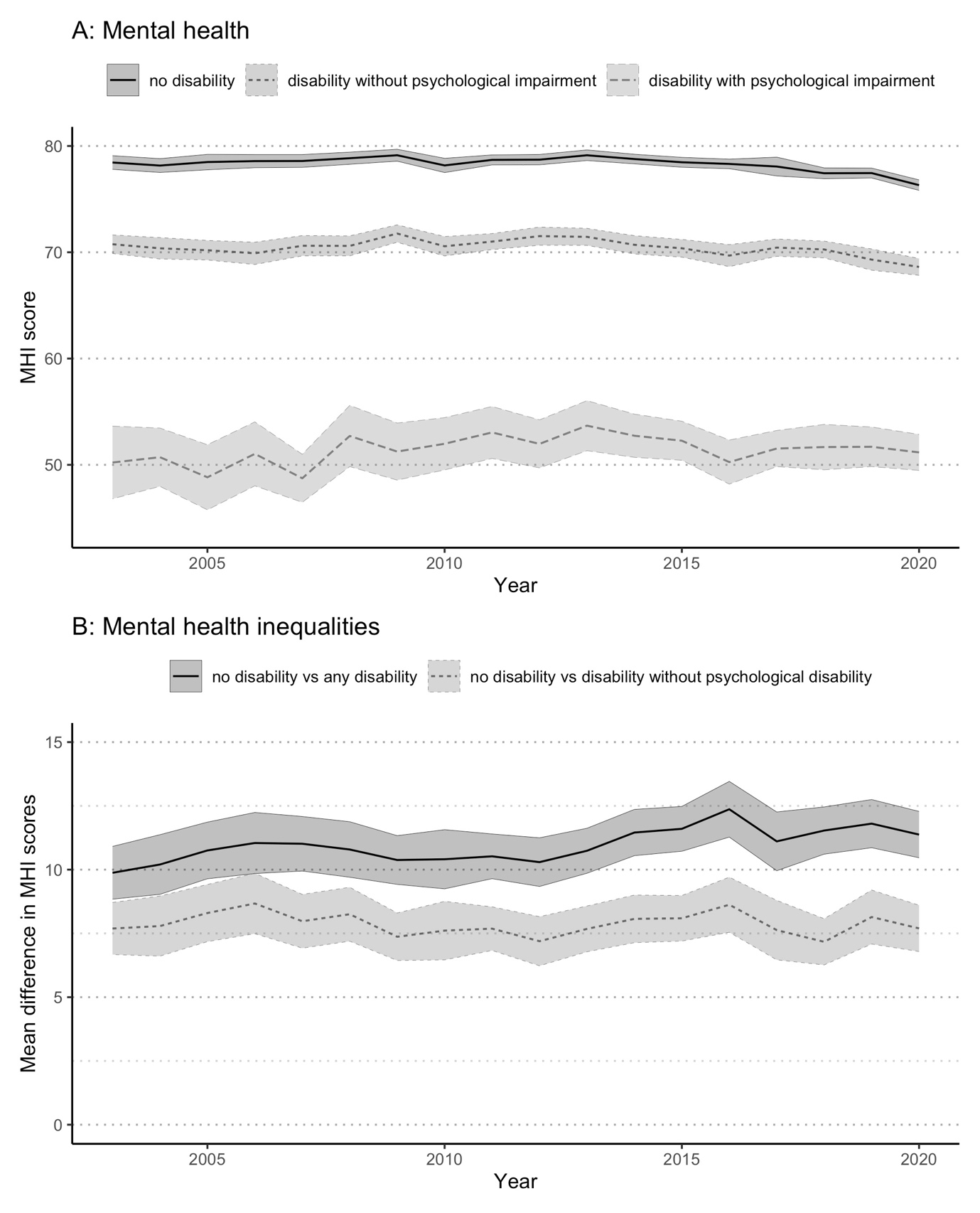


**Supplementary Figure 4.** Sensitivity analysis for people with disability who did or did not have a psychological disability, aged 15+ years. A: Age-standardised population-weighted mean MHI scores over time, with 95% confidence intervals. B: Mean difference in MHI scores over time, with 95% confidence intervals.
